# 2-Azido-1-(4-nitro­phen­yl)ethanone

**DOI:** 10.1107/S1600536812021241

**Published:** 2012-05-31

**Authors:** Sammer Yousuf, Muhammad Arshad, Hafiza Madiha Butt, Sumayya Saeed, Fatima Z. Basha

**Affiliations:** a H.E.J. Research Institute of Chemistry, International Center for Chemical and Biological Sciences, University of Karachi 75270, Pakistan; b Department of Chemistry, University of Karachi, Karachi, Pakistan

## Abstract

In the title compound, C_8_H_6_N_4_O_3_, the ketone [C—C(=O)—C] and nitro groups are tilted with respect to the benzene ring by 18.92 (6) and 24.11 (15)°, respectively. In the crystal, mol­ecules are linked into inter­woven chains running parallel to the [100] direction by C—H⋯N hydrogen bonds and weak π–π stacking inter­actions, with centroid–centroid separations of 3.897 (3) Å.

## Related literature

For the crystal structure of the related compound 2-azido-1-(4-fluoro­phen­yl)ethanone, see: Yousuf *et al.* (2012 ▶). For the biological activities of triazoles, see: Genin *et al.* (2000 ▶); Parmee *et al.* (2000 ▶); Koble *et al.* (1995 ▶); Moltzen *et al.* (1994 ▶). 
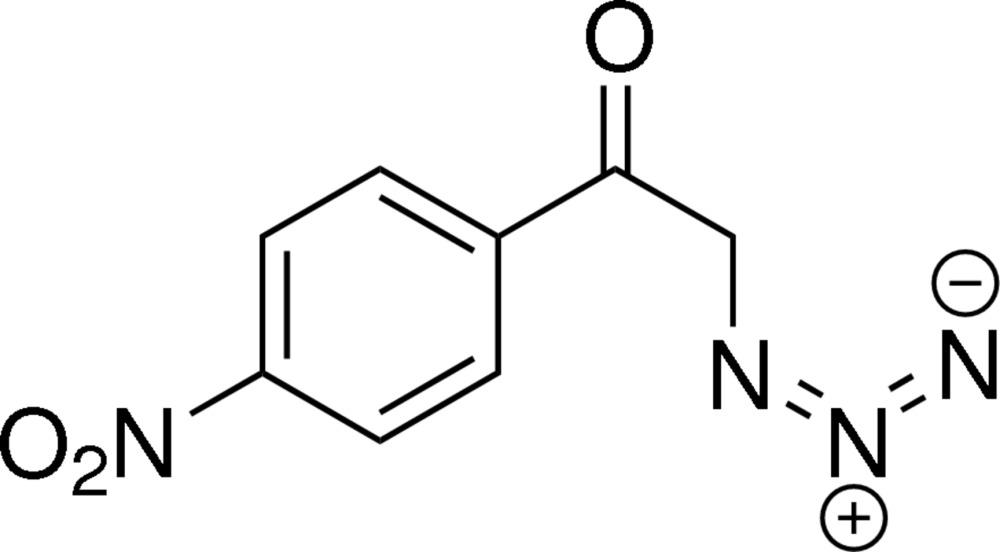


## Experimental

### 

#### Crystal data

C_8_H_6_N_4_O_3_
*M* *_r_* = 206.17
Orthorhombic, 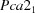

*a* = 7.6307 (5) Å
*b* = 9.5168 (6) Å
*c* = 12.4097 (8) Å
*V* = 901.19 (10) Å^3^
*Z* = 4
Mo *K*α radiation
μ = 0.12 mm^−1^
*T* = 273 K
0.50 × 0.23 × 0.11 mm

#### Data collection

Bruker SMART APEX CCD area-detector diffractometer
Absorption correction: multi-scan (*SADABS*; Bruker, 2000 ▶) *T*_min_ = 0.942, *T*_max_ = 0.987
4914 measured reflections
1649 independent reflections
1461 reflections with *I* > 2σ(*I*)
*R*_int_ = 0.020

#### Refinement

*R*[*F* ^2^ > 2σ(*F* ^2^)] = 0.032
*wR*(*F* ^2^) = 0.074
*S* = 1.06
1649 reflections
136 parameters
1 restraint
H-atom parameters constrained
Δρ_max_ = 0.11 e Å^−3^
Δρ_min_ = −0.11 e Å^−3^
Absolute structure: Flack (1983 ▶), 767 Friedel pairs
Flack parameter: 0.2 (14)

### 

Data collection: *SMART* (Bruker, 2000 ▶); cell refinement: *SAINT* (Bruker, 2000 ▶); data reduction: *SAINT*; program(s) used to solve structure: *SHELXS97* (Sheldrick, 2008 ▶); program(s) used to refine structure: *SHELXL97* (Sheldrick, 2008 ▶); molecular graphics: *SHELXTL* (Sheldrick, 2008 ▶); software used to prepare material for publication: *SHELXTL*, *PARST* (Nardelli, 1995 ▶) and *PLATON* (Spek, 2009 ▶).

## Supplementary Material

Crystal structure: contains datablock(s) global, I. DOI: 10.1107/S1600536812021241/rz2752sup1.cif

Structure factors: contains datablock(s) I. DOI: 10.1107/S1600536812021241/rz2752Isup2.hkl

Supplementary material file. DOI: 10.1107/S1600536812021241/rz2752Isup3.cml

Additional supplementary materials:  crystallographic information; 3D view; checkCIF report

## Figures and Tables

**Table 1:** Hydrogen-bond geometry (Å, °)

*D*—H⋯*A*	*D*—H	H⋯*A*	*D*⋯*A*	*D*—H⋯*A*
C8—H8*B*⋯N2^i^	0.97	2.48	3.422 (3)	165

Symmetry code: (i) 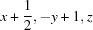.

**Fig. 1.**
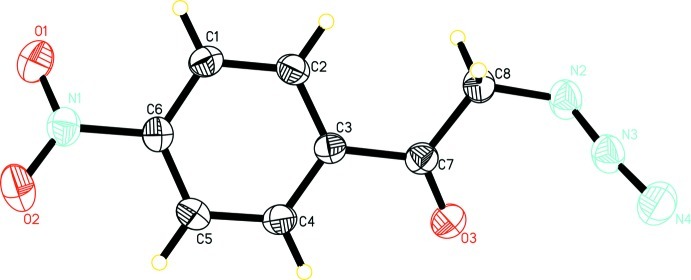
The molecular structure of the title compound with displacement ellipsoids drawn at 30% probability level.

**Fig. 2.**
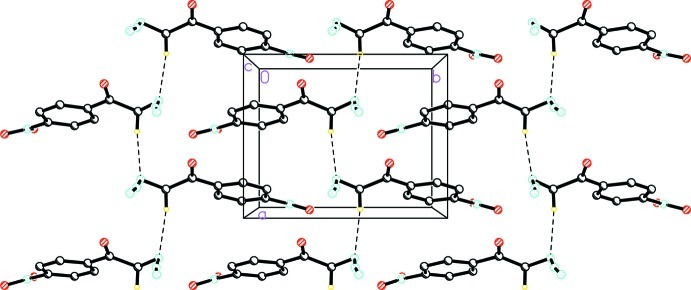
The crystal packing of the title compound. Hydrogen atoms not involved in hydrogen bonding (dashed lines) are omitted for clarity.

## supplementary crystallographic information

### Comment

The title compound was obtained as intermediate during an attempt to synthesize new triazoles, an important class of organic compounds with a wide range of biological activities (Genin *et al.*, 2000; Parmee *et al.*, 2000; Koble *et al.*, 1995; Moltzen *et al.*, 1994).

The structure of the title compound (Fig. 1) is similar to that of our recently published compound 2-azido-1-(4-fluorophenyl)ethanone (Yousuf *et al.*, 2012) with the difference that the fluorophenyl ring is replaced by a nitrobenzene ring. The benzene ring forms dihedral angles of 18.92 (6) and 24.11 (15)° with the planes through the ketone (O3/C3/C7/C8) and nitro (N1/O1/O2) groups, respectively. The azide group is not linear (N3–N2–N1 = 171.7 (2)°). The bond lengths and angle are similar to those found in the previously reported compound (Yousuf *et al.*, 2012). The crystal structure is stabilized by intermolecular C—H···N (Table 1) hydrogen bonds and π–π stacking interactions (centroid-to-centroid separations of 3.897 (3) Å) forming interwoven chains parallel to the *a* axis (Fig. 2).

### Experimental

1-(4-Nitrophenyl)ethanone (6.05 mmoL, 1.0 eq.) was dissolved in acetonitrile (18 ml) in a round bottom flask. To the stirred mixture, *p*-toluene sulphonic acid (9.08 mmoL, 1.5 eq.) and *N*-bromosuccinimide (8.48 mmol, 1.4 eq.) were added, and then heated to reflux for 1 to 1.5 h until TLC analysis showed no starting material present in the mixture. The reaction mixture was cooled to room temperature, sodium azide (18.16 mmoL, 3.0 eq.) was added and further stirred for 2 to 3 hrs followed by the addition of the ice cooled water to quench the reaction. The reaction mixture was extracted with diethyl ether (2 × 25 ml) and the combined organic layer was dried over anhydrous Na_2_SO_4_, filtered and concentrated in vacuum to get the crude product. The crude product was purified by flash silica gel chromatography (EtOAc/hexane 1/9–3/7 *v*/*v*) to afford the title compound in 70% yield. Recrystallization from ethanol afforded crystals suitable for single-crystal X-ray studies. All chemicals were purchased from Sigma-Aldrich.

### Refinement

Methylene and aromatic H atoms were positioned geometrically with C—H = 0.93–0.97 Å, and constrained to ride on their parent atoms with *U*_iso_(H) = 1.2*U*_eq_(C). 765 Friedel pairs were not merged.

### Crystal data

**Table:**

C_8_H_6_N_4_O_3_	*F*(000) = 424
*M**_r_* = 206.17	*D*_x_ = 1.520 Mg m^−^^3^
Orthorhombic, *P**c**a*2_1_	Mo *K*α radiation, λ = 0.71073 Å
Hall symbol: P 2c 2ac	Cell parameters from 1557 reflections
*a* = 7.6307 (5) Å	θ = 3.3–24.2°
*b* = 9.5168 (6) Å	µ = 0.12 mm^−^^1^
*c* = 12.4097 (8) Å	*T* = 273 K
*V* = 901.19 (10) Å^3^	Block, colourles
*Z* = 4	0.50 × 0.23 × 0.11 mm

### Data collection

**Table:**

Bruker SMART APEX CCD area-detector diffractometer	1649 independent reflections
Radiation source: fine-focus sealed tube	1461 reflections with *I* > 2σ(*I*)
Graphite monochromator	*R*_int_ = 0.020
ω scan	θ_max_ = 25.5°, θ_min_ = 2.1°
Absorption correction: multi-scan (*SADABS*; Bruker, 2000)	*h* = −9→9
*T*_min_ = 0.942, *T*_max_ = 0.987	*k* = −11→11
4914 measured reflections	*l* = −15→15

### Refinement

**Table:**

Refinement on *F*^2^	Secondary atom site location: difference Fourier map
Least-squares matrix: full	Hydrogen site location: inferred from neighbouring sites
*R*[*F*^2^ > 2σ(*F*^2^)] = 0.032	H-atom parameters constrained
*wR*(*F*^2^) = 0.074	*w* = 1/[σ^2^(*F*_o_^2^) + (0.0342*P*)^2^ + 0.0663*P*] where *P* = (*F*_o_^2^ + 2*F*_c_^2^)/3
*S* = 1.06	(Δ/σ)_max_ < 0.001
1649 reflections	Δρ_max_ = 0.11 e Å^−^^3^
136 parameters	Δρ_min_ = −0.11 e Å^−^^3^
1 restraint	Absolute structure: Flack (1983), 767 Friedel pairs
Primary atom site location: structure-invariant direct methods	Flack parameter: 0.2 (14)

### Special details

**Table:**

Geometry. All e.s.d.'s (except the e.s.d. in the dihedral angle between two l.s. planes) are estimated using the full covariance matrix. The cell e.s.d.'s are taken into account individually in the estimation of e.s.d.'s in distances, angles and torsion angles; correlations between e.s.d.'s in cell parameters are only used when they are defined by crystal symmetry. An approximate (isotropic) treatment of cell e.s.d.'s is used for estimating e.s.d.'s involving l.s. planes.
Refinement. Refinement of *F*^2^ against ALL reflections. The weighted *R*-factor *wR* and goodness of fit *S* are based on *F*^2^, conventional *R*-factors *R* are based on *F*, with *F* set to zero for negative *F*^2^. The threshold expression of *F*^2^ > σ(*F*^2^) is used only for calculating *R*-factors(gt) *etc*. and is not relevant to the choice of reflections for refinement. *R*-factors based on *F*^2^ are statistically about twice as large as those based on *F*, and *R*- factors based on ALL data will be even larger.

### Fractional atomic coordinates and isotropic or equivalent isotropic displacement parameters (Å^2^)

**Table:**

	*x*	*y*	*z*	*U*_iso_*/*U*_eq_	
O1	0.9428 (3)	1.19359 (17)	0.40028 (13)	0.0815 (5)	
O2	0.9798 (2)	1.30833 (17)	0.54808 (15)	0.0744 (5)	
O3	0.6676 (2)	0.72287 (15)	0.81581 (12)	0.0673 (4)	
N1	0.9448 (2)	1.20158 (19)	0.49879 (14)	0.0553 (5)	
N2	0.7720 (3)	0.45389 (18)	0.76819 (14)	0.0603 (5)	
N3	0.8183 (2)	0.45144 (17)	0.86298 (15)	0.0553 (4)	
N4	0.8503 (3)	0.4344 (2)	0.95022 (17)	0.0740 (6)	
C1	0.9238 (2)	0.9459 (2)	0.51250 (14)	0.0478 (5)	
H1A	0.9657	0.9388	0.4423	0.057*	
C2	0.8824 (3)	0.8273 (2)	0.57102 (15)	0.0472 (5)	
H2B	0.8991	0.7389	0.5407	0.057*	
C3	0.8160 (2)	0.83847 (19)	0.67466 (14)	0.0418 (4)	
C4	0.7943 (2)	0.9715 (2)	0.71966 (14)	0.0474 (5)	
H4A	0.7489	0.9796	0.7889	0.057*	
C5	0.8386 (2)	1.0906 (2)	0.66373 (16)	0.0482 (4)	
H5A	0.8264	1.1791	0.6946	0.058*	
C6	0.9017 (2)	1.0752 (2)	0.56038 (14)	0.0438 (4)	
C7	0.7632 (3)	0.7125 (2)	0.73869 (14)	0.0459 (4)	
C8	0.8367 (3)	0.57217 (19)	0.70483 (15)	0.0520 (5)	
H8A	0.8079	0.5563	0.6297	0.062*	
H8B	0.9634	0.5754	0.7107	0.062*	

### Atomic displacement parameters (Å^2^)

**Table:**

	*U*^11^	*U*^22^	*U*^33^	*U*^12^	*U*^13^	*U*^23^
O1	0.1121 (14)	0.0726 (11)	0.0598 (10)	−0.0001 (10)	0.0104 (9)	0.0177 (8)
O2	0.0870 (11)	0.0473 (9)	0.0889 (12)	−0.0090 (8)	−0.0091 (10)	0.0089 (9)
O3	0.0804 (11)	0.0609 (9)	0.0606 (8)	−0.0032 (7)	0.0250 (8)	0.0022 (7)
N1	0.0540 (10)	0.0514 (12)	0.0607 (12)	0.0034 (8)	−0.0015 (9)	0.0086 (9)
N2	0.0725 (12)	0.0476 (10)	0.0607 (10)	−0.0110 (9)	−0.0118 (9)	0.0040 (8)
N3	0.0606 (11)	0.0435 (9)	0.0617 (11)	−0.0068 (8)	−0.0014 (9)	0.0033 (8)
N4	0.0883 (14)	0.0733 (14)	0.0603 (11)	−0.0078 (11)	−0.0024 (11)	0.0088 (10)
C1	0.0507 (10)	0.0538 (13)	0.0390 (9)	0.0025 (9)	0.0020 (8)	−0.0012 (9)
C2	0.0520 (10)	0.0424 (11)	0.0472 (10)	0.0023 (9)	0.0000 (9)	−0.0059 (8)
C3	0.0402 (9)	0.0447 (11)	0.0406 (9)	−0.0012 (8)	−0.0020 (8)	−0.0021 (8)
C4	0.0498 (10)	0.0512 (12)	0.0413 (9)	−0.0013 (9)	0.0033 (8)	−0.0052 (9)
C5	0.0510 (10)	0.0419 (11)	0.0516 (10)	0.0002 (8)	−0.0032 (9)	−0.0080 (9)
C6	0.0399 (9)	0.0439 (11)	0.0475 (9)	−0.0005 (8)	−0.0050 (8)	0.0046 (9)
C7	0.0460 (9)	0.0490 (11)	0.0426 (9)	−0.0047 (9)	−0.0030 (9)	−0.0025 (8)
C8	0.0594 (12)	0.0456 (12)	0.0510 (10)	−0.0008 (10)	−0.0012 (10)	0.0031 (9)

### Geometric parameters (Å, º)

**Table:**

O1—N1	1.225 (2)	C2—H2B	0.9300
O2—N1	1.216 (2)	C3—C4	1.393 (3)
O3—C7	1.207 (2)	C3—C7	1.493 (3)
N1—C6	1.462 (2)	C4—C5	1.372 (3)
N2—N3	1.229 (2)	C4—H4A	0.9300
N2—C8	1.459 (2)	C5—C6	1.378 (3)
N3—N4	1.122 (2)	C5—H5A	0.9300
C1—C6	1.377 (3)	C7—C8	1.508 (3)
C1—C2	1.379 (3)	C8—H8A	0.9700
C1—H1A	0.9300	C8—H8B	0.9700
C2—C3	1.386 (2)		
			
O2—N1—O1	123.83 (18)	C3—C4—H4A	119.4
O2—N1—C6	118.28 (16)	C4—C5—C6	118.00 (18)
O1—N1—C6	117.89 (18)	C4—C5—H5A	121.0
N3—N2—C8	115.67 (17)	C6—C5—H5A	121.0
N4—N3—N2	171.7 (2)	C1—C6—C5	122.64 (18)
C6—C1—C2	118.44 (17)	C1—C6—N1	118.80 (16)
C6—C1—H1A	120.8	C5—C6—N1	118.55 (18)
C2—C1—H1A	120.8	O3—C7—C3	121.29 (19)
C1—C2—C3	120.62 (18)	O3—C7—C8	121.17 (19)
C1—C2—H2B	119.7	C3—C7—C8	117.52 (15)
C3—C2—H2B	119.7	N2—C8—C7	114.03 (16)
C2—C3—C4	119.02 (17)	N2—C8—H8A	108.7
C2—C3—C7	122.04 (17)	C7—C8—H8A	108.7
C4—C3—C7	118.93 (15)	N2—C8—H8B	108.7
C5—C4—C3	121.25 (16)	C7—C8—H8B	108.7
C5—C4—H4A	119.4	H8A—C8—H8B	107.6
			
C8—N2—N3—N4	−176.5 (17)	O2—N1—C6—C1	−156.72 (18)
C6—C1—C2—C3	1.5 (3)	O1—N1—C6—C1	23.6 (2)
C1—C2—C3—C4	−1.0 (3)	O2—N1—C6—C5	24.0 (2)
C1—C2—C3—C7	177.54 (16)	O1—N1—C6—C5	−155.73 (18)
C2—C3—C4—C5	−0.6 (3)	C2—C3—C7—O3	−161.46 (19)
C7—C3—C4—C5	−179.14 (16)	C4—C3—C7—O3	17.1 (3)
C3—C4—C5—C6	1.5 (3)	C2—C3—C7—C8	19.8 (2)
C2—C1—C6—C5	−0.6 (3)	C4—C3—C7—C8	−161.67 (16)
C2—C1—C6—N1	−179.83 (16)	N3—N2—C8—C7	−67.2 (3)
C4—C5—C6—C1	−0.9 (3)	O3—C7—C8—N2	4.1 (3)
C4—C5—C6—N1	178.33 (16)	C3—C7—C8—N2	−177.17 (16)

### Hydrogen-bond geometry (Å, º)

**Table:**

*D*—H···*A*	*D*—H	H···*A*	*D*···*A*	*D*—H···*A*
C8—H8*B*···N2^i^	0.97	2.48	3.422 (3)	165

Symmetry code: (i) *x*+1/2, −*y*+1, *z*.
